# Anticancer Effects of Fluphenazine Alone and in Combination with Cisplatin in Gastric Cancer Cells and a Mouse Xenograft Model

**DOI:** 10.3390/ph19091373

**Published:** 2026-08-31

**Authors:** Seung-Hyeon Ahn, Jihyun Shin, Hwayoung Na, Hong Kyu Lee, Kyung-Chul Choi

**Affiliations:** 1 Laboratory of Biochemistry and Immunology, College of Veterinary Medicine, Chungbuk National University, Cheongju 28644, Republic of Korea; for-happy@naver.com (S.-H.A.); 585250@naver.com (J.S.); ghkdud1994@gmail.com (H.N.); 2 Department of Companion Animal Health, College of Biomedical Science & Health, Inje University, Gimhae 50834, Republic of Korea

**Keywords:** gastric cancer, fluphenazine, cisplatin, apoptosis, female mice

## Abstract

**Background/Objectives:** Gastric cancer remains a leading cause of cancer-related mortality worldwide, highlighting the need for novel therapeutic strategies. Drug repositioning offers a cost-effective approach by identifying new therapeutic applications for clinically approved drugs. Fluphenazine (FPZ), a dopamine receptor D2 antagonist used as an antipsychotic drug, has demonstrated anticancer activity; however, its effects on gastric cancer remain unclear. **Methods:** This study investigated the anticancer effects of FPZ alone and in combination with cisplatin (DDP) in gastric cancer. **Results:** In MKN-45 cells, viability was 100%, 101.16%, 61.26%, and 53.12% in the control, FPZ, DDP, and combination groups, respectively; the corresponding values in AGS cells were 100%, 84.95%, 89.94%, and 73.94%. FPZ also inhibited migration and induced apoptosis, while combined treatment increased cytosolic and mitochondrial reactive oxygen species levels and mitochondrial stress. Furthermore, FPZ reduced IL-6 and IL-8 expression and attenuated DDP-induced inflammatory responses. In the mouse xenograft model, the tumor volumes were 1233.65, 1115.75, 768.88, and 309.44 mm^3^ in the control, FPZ, DDP, and combination groups, respectively. **Conclusions:** These findings support the potential of FPZ as a repurposed anticancer agent for gastric cancer, particularly in combination with DDP.

## 1. Introduction

Gastric cancer is the fifth most commonly diagnosed cancer and the fourth leading cause of cancer-related mortality worldwide [1]. Owing to the asymptomatic nature of early-stage gastric cancer, many patients are diagnosed at an advanced stage and require systemic treatment. Currently, chemotherapy regimens for advanced gastric cancer commonly include platinum-based agents, such as cisplatin (DDP) and oxaliplatin, in combination with fluoropyrimidines [2]. However, the clinical efficacy of conventional chemotherapy is limited by systemic toxicity and insufficient therapeutic responses in some patients. Therefore, the development of alternative therapeutic strategies that can improve treatment efficacy remains necessary.

Drug repositioning has emerged as a promising strategy for identifying new therapeutic applications for approved drugs, particularly in anticancer research [3,4]. Compared with de novo drug development, drug repositioning offers several advantages, including established clinical safety and reduced development time and costs [5]. This approach can identify anticancer activities of existing drugs through their known molecular targets or previously unrecognized mechanisms [6,7]. Several non-oncological drugs have been investigated for repurposing as anticancer agents, including metformin, an established treatment for type 2 diabetes, and disulfiram, a drug traditionally used for the management of alcohol dependence [8,9,10]. Moreover, several studies have demonstrated that repurposed drugs can enhance the anticancer activity of conventional chemotherapeutic agents when used in combination [11,12,13]. The combination of repurposed drugs with conventional chemotherapeutic agents may enhance therapeutic efficacy through the modulation of multiple cellular pathways associated with cancer progression and treatment response [14,15]. Therefore, drug repositioning, particularly in combination with established chemotherapeutic agents, may offer an efficient strategy for expanding the therapeutic options available for gastric cancer.

Recent studies have suggested that dopamine receptor D2 (DRD2) is involved in cancer progression, and increased DRD2 expression has been reported in several cancer types, including gastric cancer [16,17,18]. These findings suggest that pharmacological targeting of DRD2 may have potential as an anticancer strategy. Fluphenazine (FPZ) is a first-generation (typical) antipsychotic belonging to the phenothiazine class and exerts its principal pharmacological effects primarily through antagonism of dopamine D2 receptors [19,20]. As a phenothiazine derivative, FPZ is structurally characterized by a trifluoromethyl-substituted phenothiazine core and a piperazine-containing side chain [17]. Since its introduction into clinical practice in the late 1950s, FPZ has been used primarily for the treatment of schizophrenia and other psychotic disorders [21]. More recently, its pharmacological properties have prompted investigations into the potential application of FPZ in cancer therapy. Previous studies have demonstrated that FPZ inhibits cancer cell proliferation and migration and induces apoptosis. In addition, FPZ has been reported to impair autophagy and induce cell death under hypoxic conditions, including in three-dimensional tumor spheroid models [22,23]. These findings suggest that FPZ may exert anticancer activity through multiple cellular mechanisms, providing a rationale for evaluating its effects both alone and in combination with conventional chemotherapeutic agents. However, the anticancer effects of FPZ and its potential use in combination with conventional chemotherapeutic agents in gastric cancer remain unclear.

This study aimed to investigate the anticancer effects of FPZ alone and in combination with DDP in gastric cancer. In vitro experiments were conducted to evaluate cell viability, migration, apoptosis, mitochondrial stress, and inflammatory cytokine expression in gastric cancer cells. In addition, three-dimensional tumor spheroid and in vivo mouse xenograft models were used to evaluate the antitumor effects of FPZ alone and in combination with DDP. Through in vitro and in vivo studies, we evaluated the anticancer effects of FPZ in gastric cancer and its potential to enhance the antitumor activity of DDP.

## 2. Results

### 2.1. FPZ Decreases Cell Viability in Gastric Cancer Cell Lines and Enhances the Cytotoxic Effect of DDP

**Figure 1:**
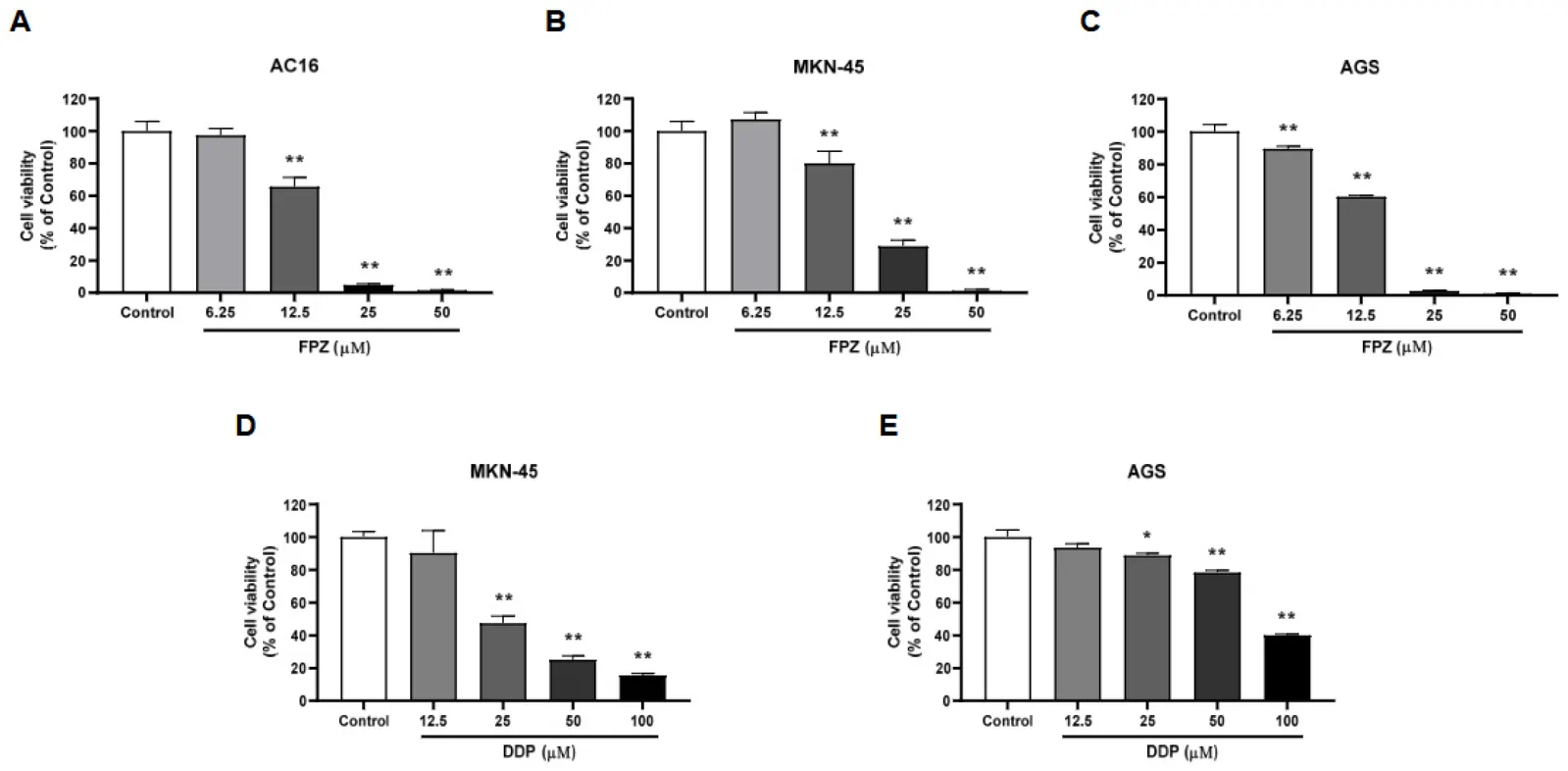
Cytotoxic effects of FPZ and DDP in gastric cancer cell lines. (**A**) Cytotoxicity of FPZ in AC16 cells determined by WST assay. (**B**,**C**) Cytotoxicity of FPZ in MKN-45 and AGS gastric cancer cell lines, respectively, as determined by WST assay. (**D**,**E**) Cytotoxicity of DDP in MKN-45 and AGS gastric cancer cell lines, respectively, as determined by WST assay. Data are expressed as the mean ± SD from three biological replicates (*n* = 3). For (**A**–**E**), five groups were analyzed using ordinary one-way ANOVA followed by Dunnett’s multiple comparisons test, with the Control group as the reference; * *p* < 0.05 and ** *p* < 0.01 vs. Control. FPZ, fluphenazine; DDP, cisplatin.

**Figure 2:**
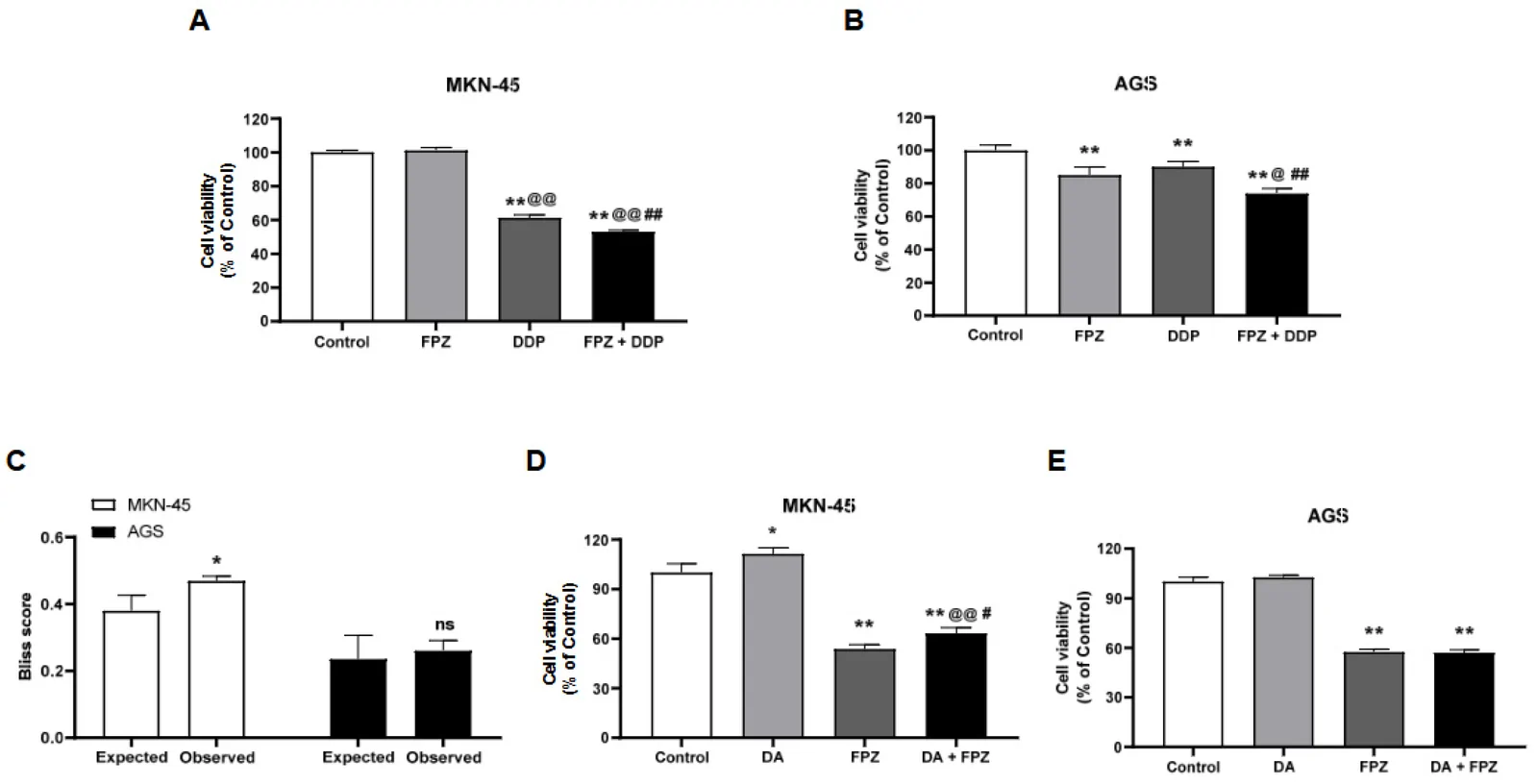
FPZ enhances the anticancer effects of DDP in gastric cancer cell lines. (**A**,**B**) Changes in cell viability following combined treatment with FPZ and DDP in MKN-45 and AGS gastric cancer cell lines, respectively. (**C**) Evaluation of the combined effects of FPZ and DDP co-treatment using the Bliss model. (**D**,**E**) DA dependency of the effects of FPZ in MKN-45 and AGS gastric cancer cell lines, respectively. Data are expressed as the mean ± SD from three biological replicates (*n* = 3). For (**A**,**B**), four groups were analyzed using ordinary two-way ANOVA followed by Dunnett’s multiple comparisons test, with Control, FPZ, or DDP used as the reference group as indicated; ** *p* < 0.01 vs. Control, ^@^ *p* < 0.05 and ^@@^ *p* < 0.01 vs. FPZ, and ^##^ *p* < 0.01 vs. DDP. For (**C**), the expected and observed Bliss scores from three biological replicates (*n* = 3) were compared using an unpaired *t*-test; * *p* < 0.05 vs. Expected; ns, not significant. For (**D**), four groups were analyzed using ordinary one-way ANOVA followed by Dunnett’s multiple comparisons test, with Control, DA, or FPZ used as the reference group as indicated; * *p* < 0.05 and ** *p* < 0.01 vs. Control, ^@@^ *p* < 0.01 vs. DA, and ^#^ *p* < 0.05 vs. FPZ. For (**E**), four groups were analyzed using ordinary two-way ANOVA followed by Dunnett’s multiple comparisons test, with the Control group as the reference; ** *p* < 0.01 vs. Control. FPZ, fluphenazine; DDP, cisplatin; DA, dopamine.

Previous studies have demonstrated the anticancer effects of FPZ across various cancer types [17]. To determine a sub-toxic concentration, the cytotoxicity of FPZ was assessed in AC16 human cardiomyocyte cells using a WST assay. FPZ exhibited minimal cytotoxicity up to a concentration of 6 μM (Figure 1A). FPZ treatment resulted in a dose-dependent reduction in cell viability in both MKN-45 and AGS gastric cancer cell lines (Figure 1B,C). To establish the optimal concentration for combination therapy, the IC_50_ values of DDP were determined for both gastric cancer cell lines using a WST assay. The concentrations corresponding to 50% of the IC_50_ of DDP were approximately 19 μM in MKN-45 cells and 39.5 μM in AGS cells (Figure 1D,E). To avoid excessive cytotoxicity from DDP, FPZ (6 μM) was combined with a 0.5× IC_50_ concentration of DDP for each cell line. The effects of the combination treatment were assessed using a WST assay. The combined treatment with FPZ and DDP significantly reduced cell viability compared with the control and monotherapy treatment groups (Figure 2A,B). To evaluate the interaction between the two drugs, the Bliss independence model was applied. The combination of FPZ and DDP exhibited a synergistic effect in MKN-45 cells and an additive effect in AGS cells (Figure 2C). To determine whether the difference in the anticancer effects of FPZ between MKN-45 and AGS cells was due to differences in dopamine (DA) sensitivity, cell viability was assessed via a WST assay after treatment with 20 μM DA. The results showed that the DA treatment increased cell viability in the MKN-45 cells and reduced the anticancer effect of FPZ. In contrast, no significant changes were observed in AGS cells regardless of DA treatment (Figure 2D,E). These results suggest that FPZ not only reduces the viability of gastric cancer cells when used alone but also enhances the cytotoxic effect of DDP, with the magnitude of the combined effect differing between the two cell lines based on DA sensitivity.

### 2.2. Treatment with FPZ Alone and in Combination with DDP Inhibits the Migration of Gastric Cancer Cell Lines

**Figure 3:**
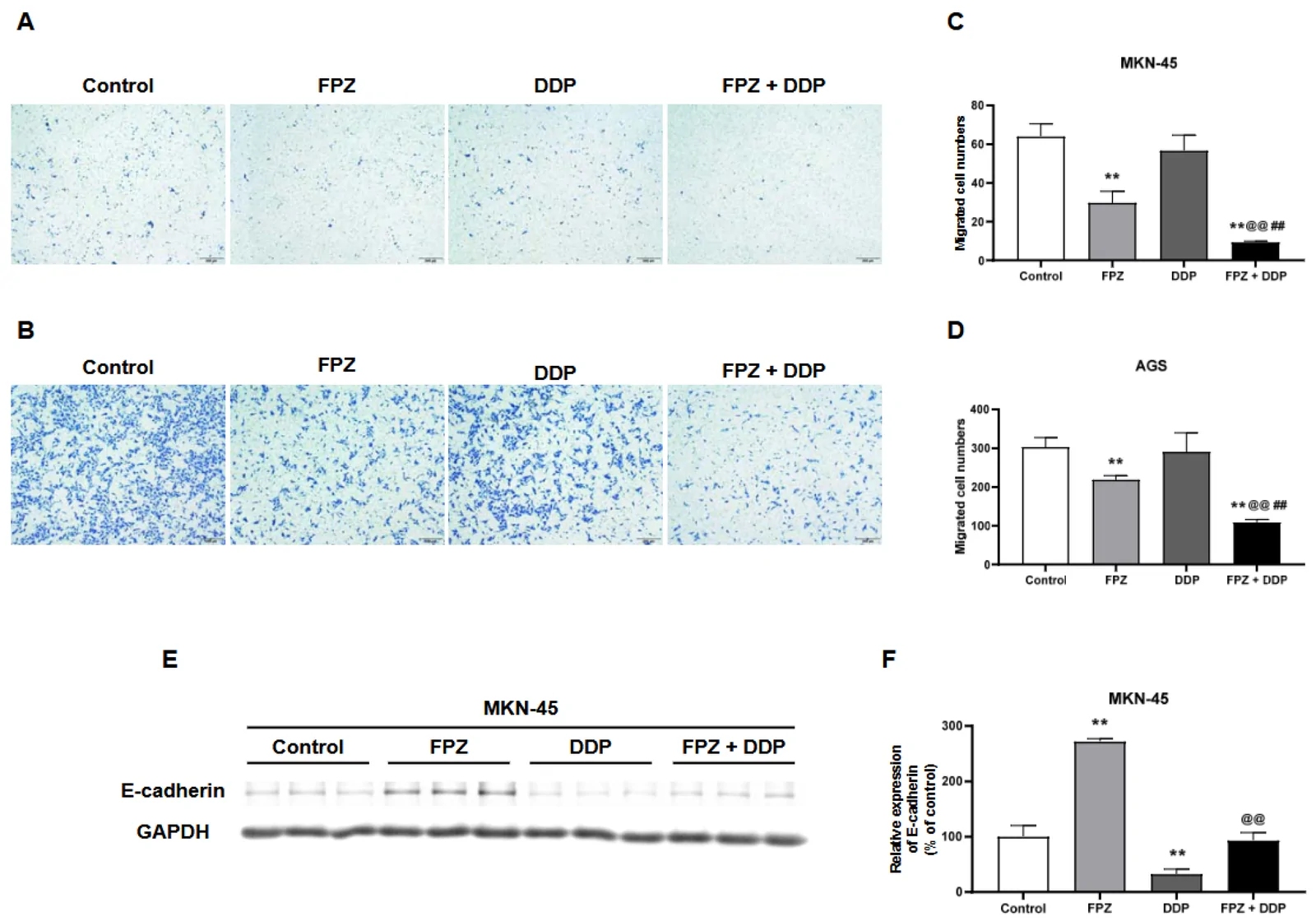
Treatment with FPZ alone and in combination with DDP inhibits the migration of gastric cancer cell lines. (**A**,**B**) Representative images of the Transwell assay. (**C**,**D**) Quantitative analysis of Transwell assay results. (**E**) Representative Western blot images. (**F**) Quantification of Western blot band intensities. Data are expressed as the mean ± SD from three biological replicates (*n* = 3). Two factors (FPZ and DDP treatment) were analyzed using two-way ANOVA followed by Dunnett’s multiple comparisons test. ** *p* < 0.01 vs. Control; ^@@^ *p* < 0.01 vs. FPZ; ^##^ *p* < 0.01 vs. DDP. Bar = 200 μm. FPZ, fluphenazine; DDP, cisplatin.

Cancer progression through metastasis significantly affects prognosis and suppression of the migratory capacity of cancer cells is a critical challenge for effective cancer treatment [24,25]. To investigate whether FPZ alone or in combination with DDP modulated the migratory capacity of gastric cancer cells, transwell migration assays were performed (Figure 3A,B). While DDP monotherapy showed no significant effect on cell migration, the FPZ monotherapy significantly inhibited migratory potential. Furthermore, the combination of FPZ and DDP exerted a more potent inhibitory effect than either single agent alone (Figure 3C,D). Since cancer cells may acquire migratory ability through a well-characterized biochemical process known as EMT [26,27], we examined expression of E-cadherin, an EMT marker, in MKN-45 cells after treatment (Figure 3E). FPZ monotherapy increased E-cadherin expression, and in the combination group, FPZ restored the DDP-induced reduction in E-cadherin levels (Figure 3F). Collectively, these findings suggest that FPZ may suppress the migratory ability of gastric cancer cells and potentially attenuate DDP-induced EMT.

### 2.3. Combined Treatment with FPZ and DDP Increases Apoptosis in Gastric Cancer Cell Lines More than Either Agent Alone

**Figure 4:**
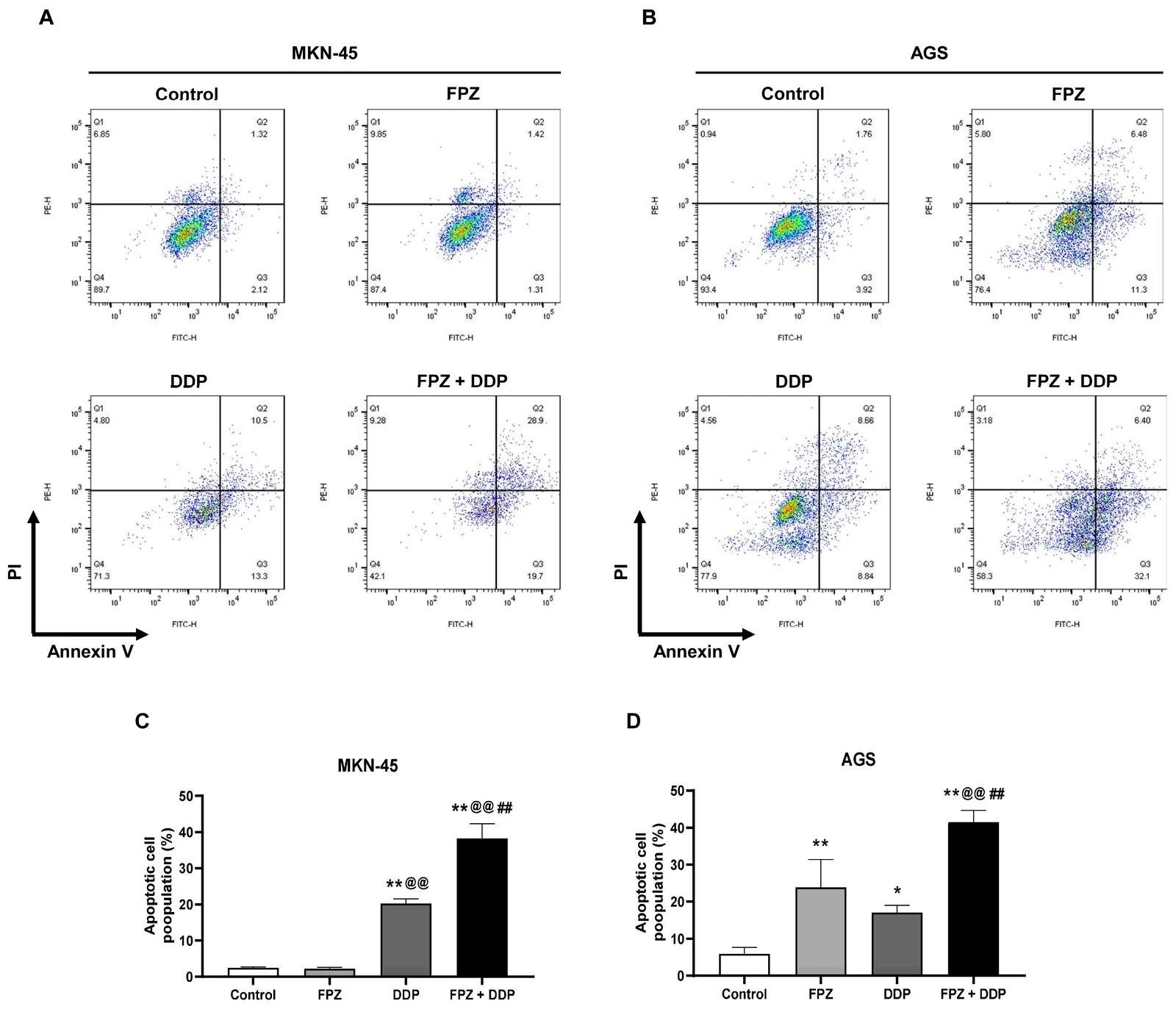
Combined treatment with FPZ and DDP increases apoptosis in gastric cancer cell lines more than either chemical alone. (**A**,**B**) Representative flow cytometric dot plots of Annexin V/PI staining. (**C**,**D**) Quantitative analysis of apoptotic cell populations determined by Annexin V/PI staining. Data are expressed as the mean ± SD from three biological replicates (*n* = 3). Four experimental groups (Control, FPZ, DDP, and FPZ + DDP), comprising two independent variables (FPZ treatment and DDP treatment), were analyzed using two-way ANOVA followed by Dunnett’s multiple comparisons test, with Control, FPZ, or DDP used as the reference group as indicated; * *p* < 0.05 and ** *p* < 0.01 vs. Control; ^@@^ *p* < 0.01 vs. FPZ; ^##^ *p* < 0.01 vs. DDP. FPZ, fluphenazine; DDP, cisplatin.

**Figure 5:**
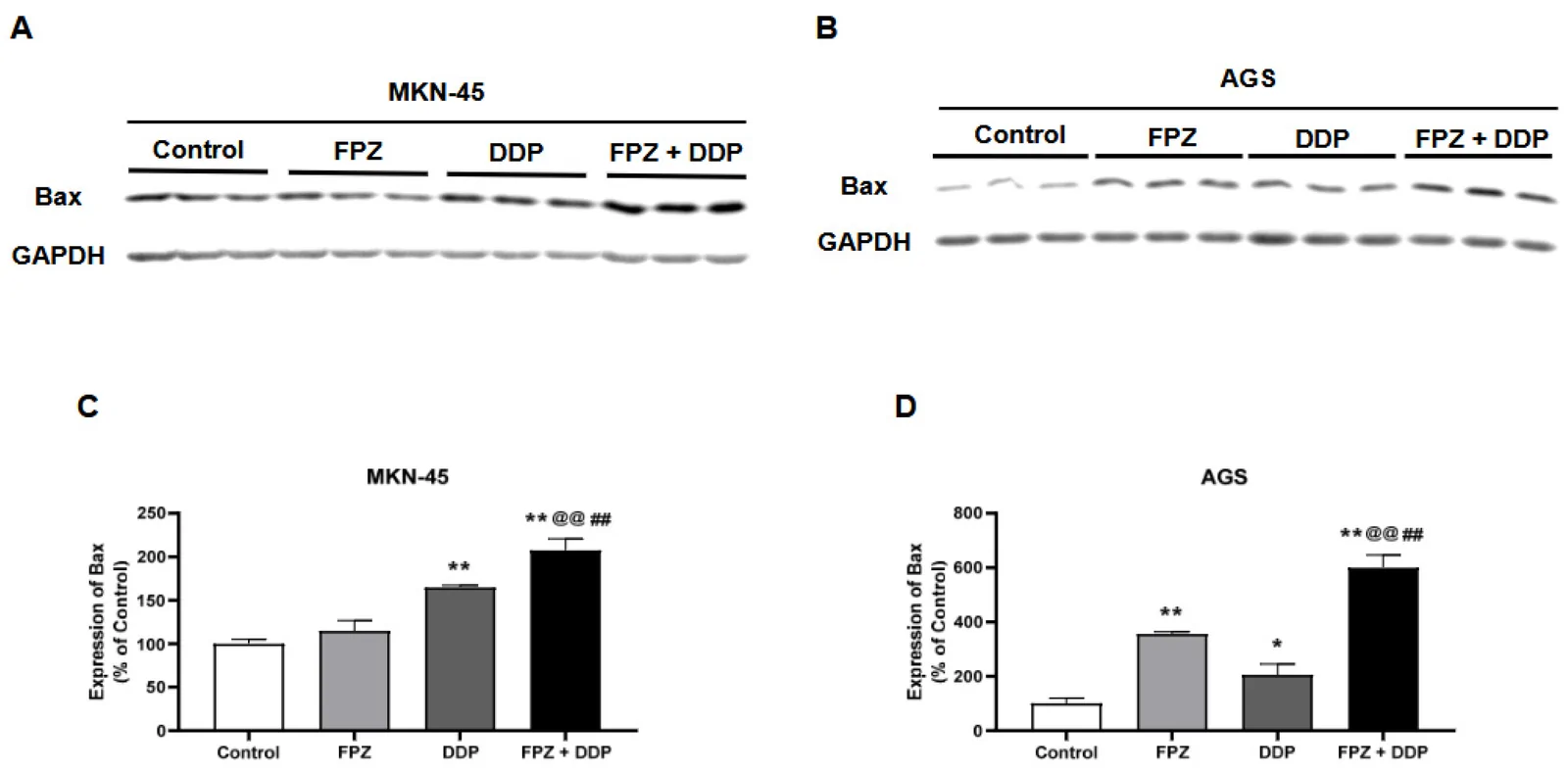
Combined treatment with FPZ and DDP increases Bax protein expression in gastric cancer cell lines. (**A**,**B**) Changes in Bax protein expression assessed by Western blot. (**C**,**D**) Quantification of Western blot band intensities. Data are expressed as the mean ± SD from three biological replicates (*n* = 3). Four experimental groups (Control, FPZ, DDP, and FPZ + DDP), comprising two independent variables (FPZ treatment and DDP treatment), were analyzed using two-way ANOVA followed by Dunnett’s multiple comparisons test, with Control, FPZ, or DDP used as the reference group as indicated; * *p* < 0.05 and ** *p* < 0.01 vs. Control; ^@@^ *p* < 0.01 vs. FPZ; ^##^ *p* < 0.01 vs. DDP. FPZ, fluphenazine; DDP, cisplatin.

Based on the intrinsic apoptosis-inducing effect of DDP [28,29], Annexin V and PI staining were performed to determine whether FPZ could potentiate the pro-apoptotic activity of DDP (Figure 4A,B). FPZ monotherapy resulted in a significant increase in the apoptotic cell population in AGS cells. Notably, combined treatment with FPZ and DDP induced significantly higher levels of apoptosis than monotherapy with either agent in both MKN-45 and AGS cells (Figure 4C,D). The expression of Bax protein, a pro-apoptotic marker, was analyzed by Western blotting (Figure 5A,B). Combined treatment with FPZ and DDP enhanced Bax expression in both MKN-45 and AGS cells compared to treatment with the individual agents alone (Figure 5C,D). These results indicate that FPZ induces apoptosis and potentiates the pro-apoptotic effects of DDP in gastric cancer cell lines.

### 2.4. Co-Treatment with FPZ and DDP Induces a Greater Increase in Intracellular ROS in Gastric Cancer Cells than Either Agent Alone

**Figure 6:**
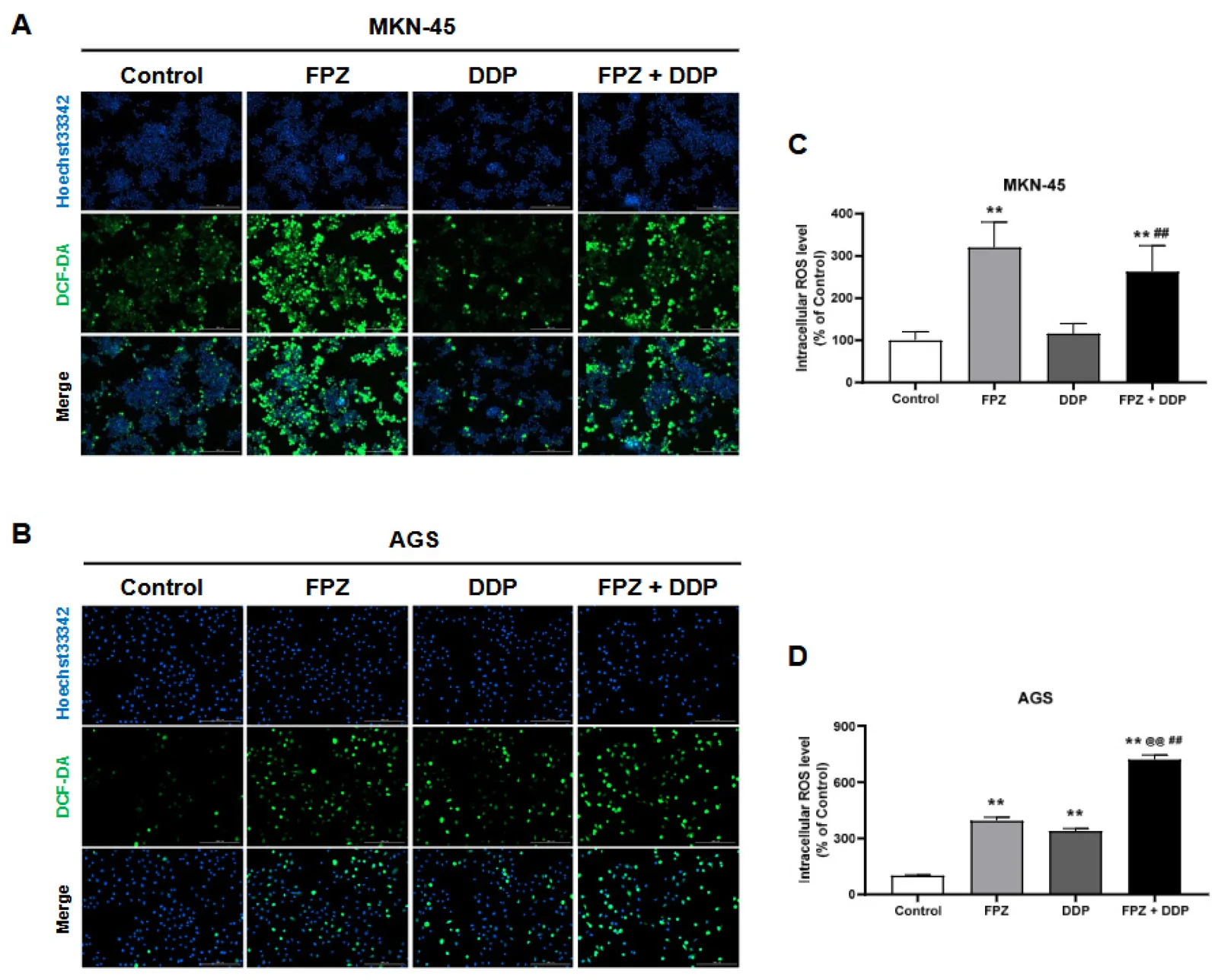
Co-treatment with FPZ and DDP induces a greater increase in intracellular ROS levels in gastric cancer cells compared with either agent alone. (**A**,**B**) Representative images of DCF-DA staining. (**C**,**D**) Quantitative analysis of DCF-DA fluorescence intensity normalized to cell number. Data are expressed as the mean ± SD from three biological replicates (*n* = 3). Four experimental groups (Control, FPZ, DDP, and FPZ + DDP), comprising two independent variables (FPZ treatment and DDP treatment), were analyzed using two-way ANOVA followed by Dunnett’s multiple comparisons test, with Control, FPZ, or DDP used as the reference group as indicated; ** *p* < 0.01 vs. Control; ^@@^ *p* < 0.01 vs. FPZ; ^##^ *p* < 0.01 vs. DDP. Bar = 200 μm. FPZ, fluphenazine; DDP, cisplatin.

Elevated intracellular ROS levels play a critical role as a trigger of intrinsic apoptosis and is widely recognized as a key indicator of cellular stress [30,31]. Based on this evidence, we investigated whether FPZ induces cytosolic ROS production in gastric cancer cell lines and whether the combination of FPZ and DDP enhances ROS production compared to either drug alone using DCF-DA staining (Figure 6A,B). Both FPZ and DDP monotherapy significantly increased intracellular ROS levels compared with the control group. The combination of FPZ and DDP induced a significantly higher increase in ROS levels than either drug alone (Figure 6C,D). These findings suggest that FPZ induces cytosolic ROS generation and cellular stress, which are further potentiated by co-treatment with DDP in gastric cancer cells.

### 2.5. FPZ Treatment Alone and in Combination with DDP Induces Mitochondrial Stress in Gastric Cancer Cell Lines

**Figure 7:**
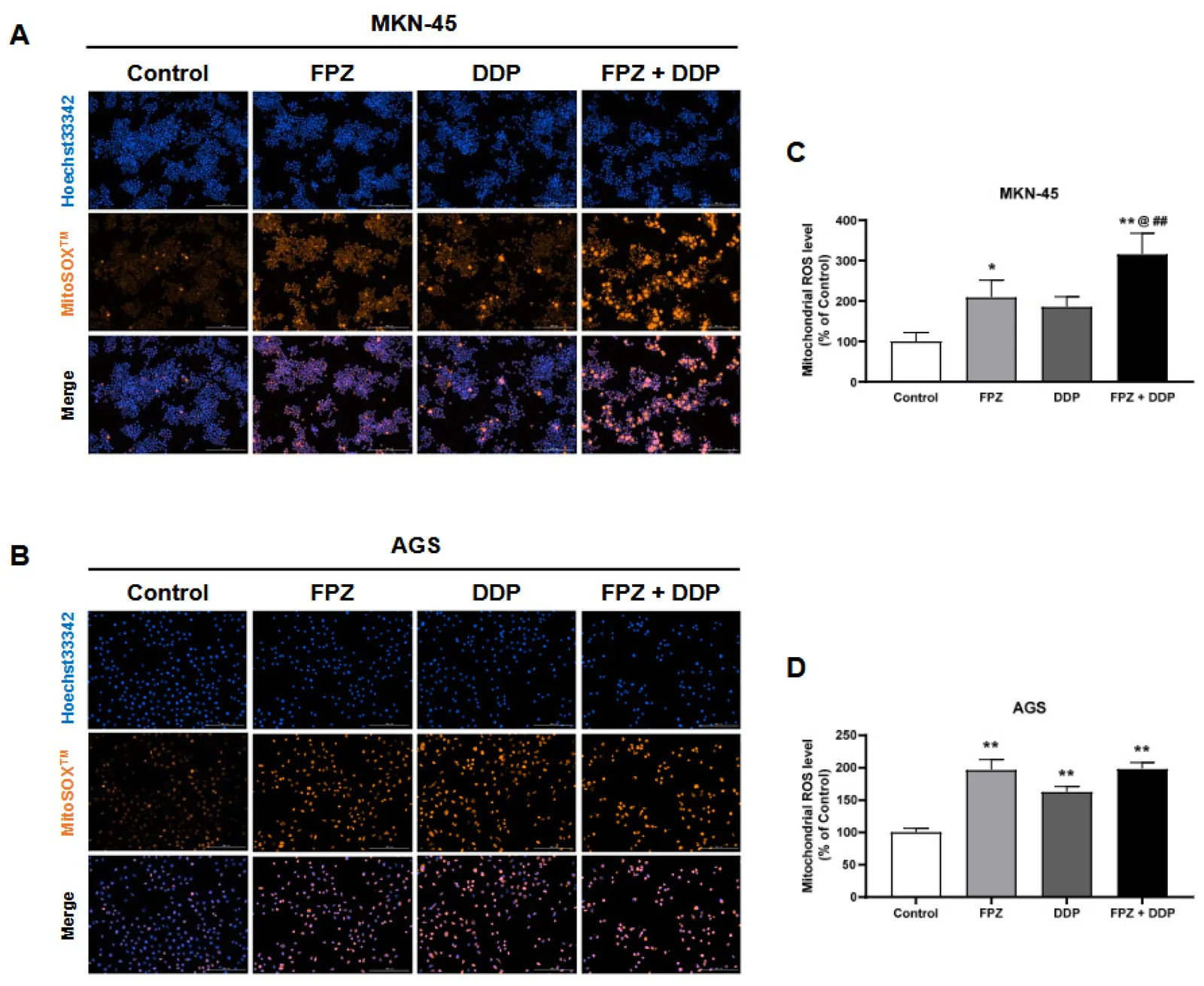
FPZ treatment alone and in combination with DDP induces mitochondrial stress in gastric cancer cell lines. (**A**,**B**) Representative images of MitoSOX™ staining. (**C**,**D**) Quantitative analysis of MitoSOX™ fluorescence intensity normalized to cell number. Data are expressed as the mean ± SD from three biological replicates (*n* = 3). Four experimental groups (Control, FPZ, DDP, and FPZ + DDP), comprising two independent variables (FPZ treatment and DDP treatment), were analyzed using two-way ANOVA followed by Dunnett’s multiple comparisons test, with Control, FPZ, or DDP used as the reference group as indicated; * *p* < 0.05 and ** *p* < 0.01 vs. Control; ^@^ *p* < 0.05 vs. FPZ; ^##^ *p* < 0.01 vs. DDP. Bar = 200 μm. FPZ, fluphenazine; DDP, cisplatin.

Accumulation of mitochondrial ROS is recognized as a pivotal factor in inducing mitochondrial stress and triggering intrinsic apoptosis [32,33]. We investigated the alterations in mitochondrial ROS levels in gastric cancer cells following FPZ monotherapy and its co-treatment with DDP, using MitoSOX™ staining (Figure 7A,B). In MKN-45 cells, treatment with FPZ or DDP alone significantly increased mitochondrial ROS levels compared with the control group, and the combined treatment induced a markedly greater increase than either agent alone (Figure 7C). A similar tendency was observed in AGS cells, in which FPZ or DDP alone, or their combination, increased mitochondrial ROS levels, although the magnitude of these changes was less pronounced than that observed in MKN-45 cells (Figure 7D). These findings suggest that FPZ induces mitochondrial stress, and the combination of FPZ and DDP exacerbates mitochondrial dysfunction, thereby potentiating intrinsic apoptotic signaling in gastric cancer cells.

### 2.6. Co-Treatment of FPZ and DDP Induces the Cell Death of Gastric Cancer Cell Lines in a Drug-Resistant Environment

**Figure 8:**
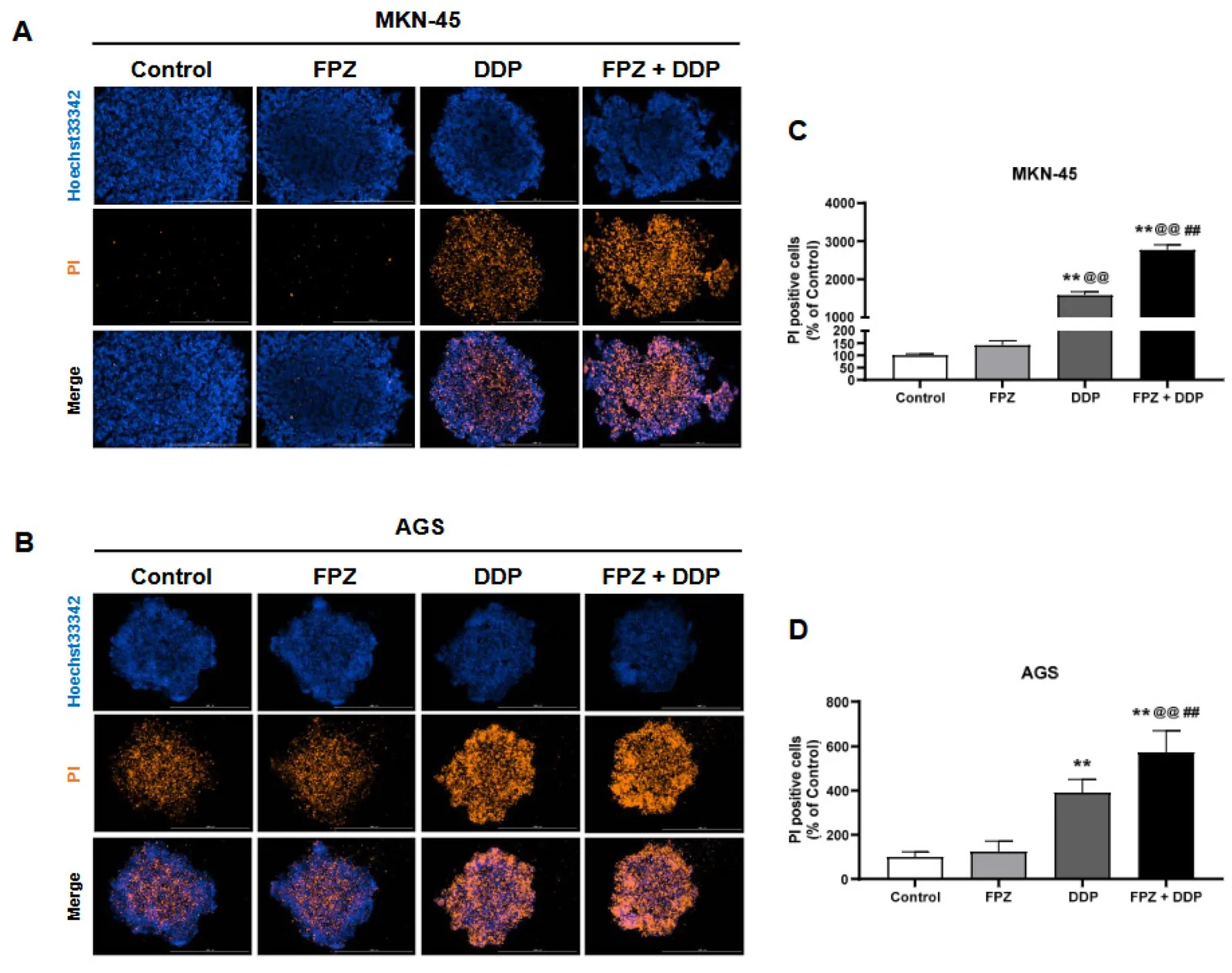
The co-treatment of FPZ and DDP induces cell death of gastric cancer cell lines in a drug-resistant environment. (**A**,**B**) Representative images of Hoechst 33342 and PI double staining of spheroids. (**C**,**D**) Quantification of PI fluorescence intensity relative to spheroid area. Data are expressed as the mean ± SD from six biological replicates (*n* = 6). Normality was assessed using the Shapiro–Wilk test prior to parametric statistical analysis. Four experimental groups (Control, FPZ, DDP, and FPZ + DDP), comprising two independent variables (FPZ treatment and DDP treatment), were analyzed using two-way ANOVA followed by Dunnett’s multiple comparisons test, with Control, FPZ, or DDP used as the reference group as indicated; ** *p* < 0.01 vs. Control; ^@@^ *p* < 0.01 vs. FPZ; ^##^ *p* < 0.01 vs. DDP. Bar = 1000 μm. FPZ, fluphenazine; DDP, cisplatin.

Since tumors formed in vivo possess a complex three-dimensional (3D) architecture, validating anticancer efficacy in both two-dimensional (2D) and 3D culture systems is essential for a more comprehensive assessment [34,35]. In this study, we evaluated the effects of FPZ and DDP alone and in combination on gastric cancer cells in a 3D model using Hoechst 33342 and PI double staining (Figure 8A,B). In contrast to the 2D environment, FPZ monotherapy did not exhibit a detectable effect in the 3D model. However, co-treatment with FPZ and DDP induced significantly greater cell death than monotherapy in the 3D model (Figure 8C,D). These findings suggest that the combination of FPZ and DDP exerts enhanced anticancer effects under both 2D and drug-resistant 3D culture conditions.

### 2.7. FPZ Attenuates DDP-Induced Pro-Inflammatory Cytokine Expression

**Figure 9:**
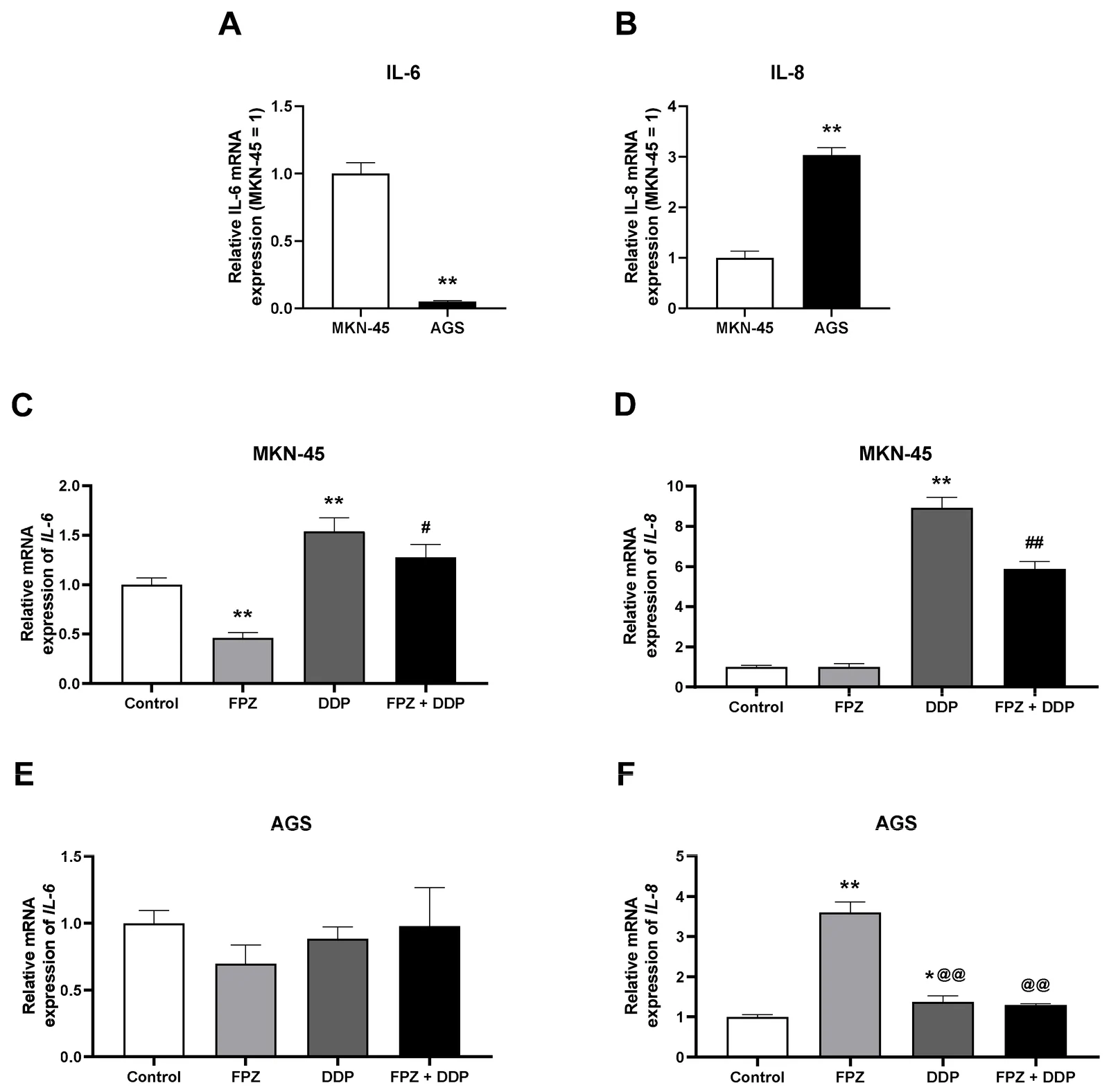
FPZ inhibits the production of inflammatory cytokines induced by DDP. (**A**) Basal IL-6 mRNA expression levels in gastric cancer cell lines. (**B**) Basal IL-8 mRNA expression levels in gastric cancer cell lines. (**C**,**D**) Changes in IL-6 and IL-8 mRNA expression in MKN-45 cells. (**E**,**F**) Changes in IL-6 and IL-8 mRNA expression in AGS cells. Data are expressed as the mean ± SD from three biological replicates (*n* = 3). For (**A**,**B**), two experimental groups (MKN-45 and AGS) were compared using an unpaired *t*-test. For (**C**–**F**), four experimental groups (Control, FPZ, DDP, and FPZ + DDP), comprising two independent variables (FPZ treatment and DDP treatment), were analyzed using two-way ANOVA followed by Dunnett’s multiple comparisons test, with Control, FPZ, or DDP used as the reference group as indicated; * *p* < 0.05 and ** *p* < 0.01 vs. Control; ^@@^ *p* < 0.01 vs. FPZ; ^#^ *p* < 0.05 and ^##^ *p* < 0.01 vs. DDP. FPZ, fluphenazine; DDP, cisplatin.

Cancer cells are characterized not only by their ability to metastasize and proliferate, but also by their ability to induce an inflammatory tumor microenvironment [36,37]. Some studies have reported that drug resistance in gastric cancer may be attributed to inflammation [38,39]. In this study, we analyzed the mRNA expression levels of pro-inflammatory cytokines in gastric cancer cell lines. We found that IL-6 mRNA expression was high in MKN-45 cells, while IL-8 mRNA expression was relatively high in AGS cells (Figure 9A,B). Based on these results, we evaluated whether FPZ treatment could modulate the mRNA expression of these cytokines in gastric cancer cells and potentially attenuate DDP-induced inflammatory responses. In MKN-45 cells, FPZ significantly inhibited the mRNA expression of inflammatory cytokines IL-6 and IL-8, and attenuated the DDP-induced upregulation of IL-6 and IL-8 mRNA expression (Figure 9C,D). In AGS cells, no significant changes were observed in the mRNA levels of the proinflammatory cytokine IL-6. However, IL-8 mRNA expression was increased in the FPZ-alone treatment group, and this effect was significantly mitigated when combined with DDP (Figure 9E,F). Collectively, these findings suggest that FPZ modulates pro-inflammatory cytokine expression and may attenuate DDP-induced inflammatory responses in gastric cancer cells.

### 2.8. FPZ Potentiates the In Vivo Antitumor Efficacy of DDP in an MKN-45 Xenograft Mouse Model

**Figure 10:**
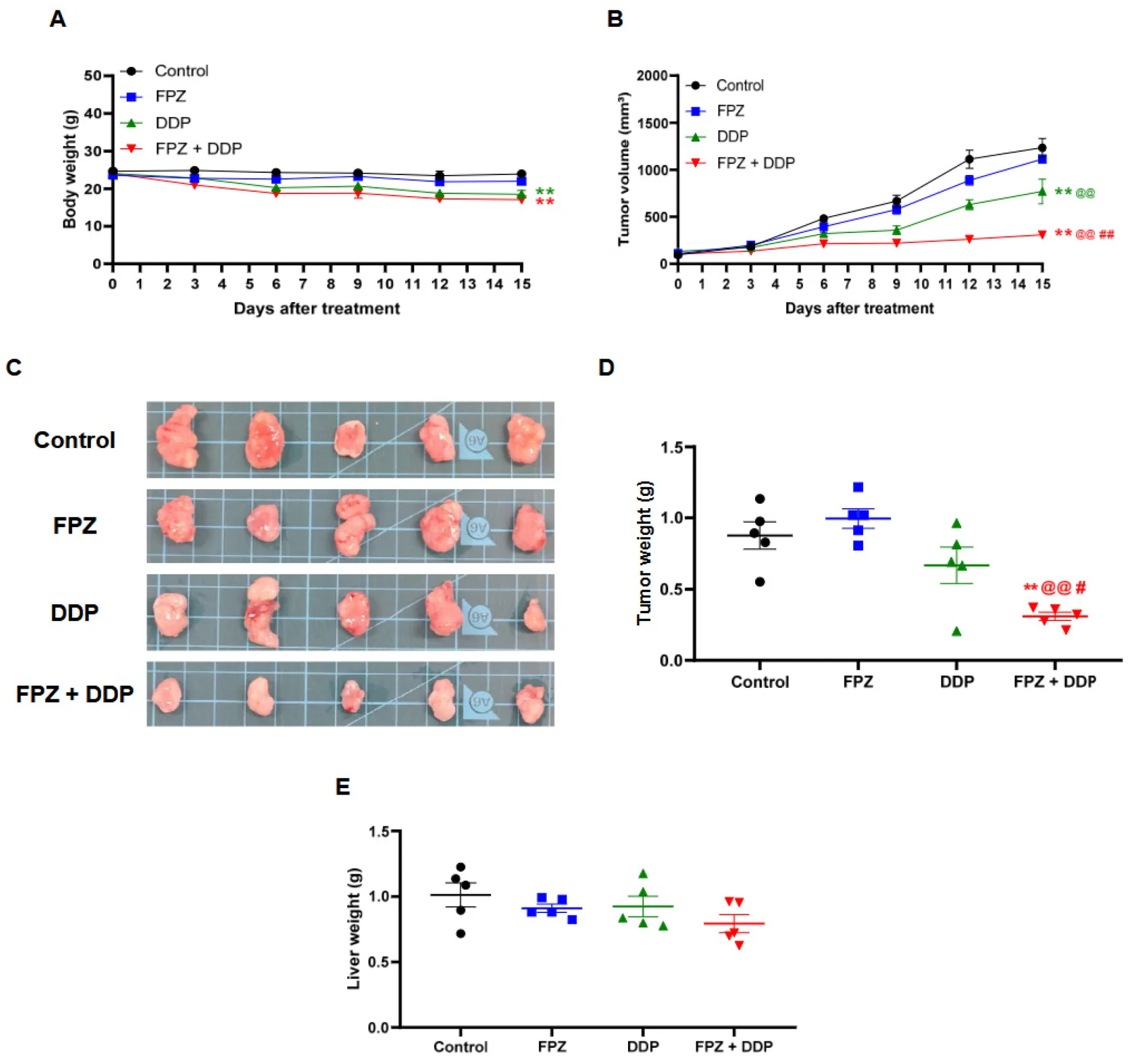
Combination treatment with FPZ and DDP enhances antitumor effects in a xenograft model of gastric cancer. (**A**) Changes in mouse body weight. (**B**) Tumor volume measurement. (**C**) Representative images of excised tumors. (**D**) Tumor weight measurement. (**E**) Liver weight measurement. Data are expressed as the mean ± SEM (*n* = 5 mice per group). Normality was assessed using the Shapiro–Wilk test prior to parametric statistical analysis. Four experimental groups (Control, FPZ, DDP, and FPZ + DDP), comprising two independent variables (FPZ treatment and DDP treatment), were analyzed using two-way ANOVA followed by Dunnett’s multiple comparisons test, with Control, FPZ, or DDP used as the reference group as indicated; ** *p* < 0.01 vs. Control; ^@@^ *p* < 0.01 vs. FPZ; ^#^ *p* < 0.05 and ^##^ *p* < 0.01 vs. DDP. FPZ, fluphenazine; DDP, cisplatin.

To validate the in vivo anticancer efficacy of FPZ, we established a xenograft mouse model by subcutaneously implanting MKN-45 cells. In the FPZ single-treatment group, no significant weight loss was observed compared with the control group, whereas significant weight loss was observed in the DDP alone and combination treatment groups (Figure 10A). While FPZ monotherapy did not significantly reduce tumor volume, both DDP monotherapy and the combination treatment resulted in a significant decrease in tumor volume. Notably, the co-treated group exhibited a markedly more potent inhibitory effect compared to either monotherapy (Figure 10B). In addition, tumor tissues were harvested to assess changes in tumor weight (Figure 10C). Tumor weight in the single-treatment groups did not differ significantly from that in the control group, whereas the combination-treatment group exhibited a significant reduction in tumor weight compared with the control group and each single-treatment group (Figure 10D). Liver weight was measured to evaluate the potential in vivo toxicity of FPZ, and no significant differences were observed among the groups (Figure 10E). Collectively, these findings suggest that FPZ potentiates the antitumor efficacy of DDP in vivo, with no significant differences in liver weight observed among the treatment groups.

## 3. Discussion

The repurposing strategy using older drugs offers a significant advantage by utilizing therapeutic agents with established safety profiles, thereby potentially reducing the time and cost required for drug development [40]. FPZ, a conventional antipsychotic and potent DRD2 antagonist, has recently emerged as a promising candidate for drug repurposing in oncology [17]. Previous studies have reported that the expression of DRD2 increases with gastric cancer progression and suggest that DRD2 plays an important role in gastric tumorigenesis [16,18]. Accordingly, the present study investigated the anticancer efficacy of FPZ, with particular emphasis on its potential to enhance the effects of DDP and the cellular mechanisms associated with this combination in gastric cancer.

Our findings demonstrated that FPZ at a concentration of 6 μM did not exert cytotoxic effects in AC16 normal cardiomyocytes while enhancing the anticancer effects of DDP in gastric cancer cell lines. These findings support the potential of FPZ as an adjuvant to conventional chemotherapeutic agents. Previous studies have primarily focused on the anticancer activity of FPZ as a single agent, whereas studies evaluating FPZ in combination with conventional chemotherapeutic agents remain relatively limited [23,41,42]. In contrast, the current results underscore the potential of FPZ as a potent adjuvant that enhances the anticancer efficacy of DDP, thus distinguishing the current study from prior reports. Additionally, this study indirectly investigated the potential role of DA signaling in the anticancer effects of FPZ by assessing whether DA treatment attenuated the FPZ-induced reduction in cell viability. The findings suggest that MKN-45 cells may be relatively more susceptible to DA-mediated modulation, whereas AGS cells appear to exhibit comparatively lower sensitivity to DA. Nevertheless, the precise contribution of DRD2-associated signaling to the anticancer activity of FPZ remains unclear. Therefore, further investigations are warranted to compare basal DRD2 expression levels among gastric cancer cell lines and to evaluate the effects of DA treatment across a broader concentration range.

Previous studies have demonstrated that DRD2 signaling is closely associated with EMT and that DRD2 antagonists suppress the migratory capacity of various cancer cells [43,44]. The current study demonstrated that FPZ significantly inhibited the migratory ability of gastric cancer cells, and co-treatment with DDP further suppressed cell motility. These findings suggest that FPZ exerts anti-migratory effects and may modulate EMT-related changes induced by DDP. In AGS cells, E-cadherin expression was undetectable, which may be attributable to the intrinsically low basal expression of this protein in this cell line [45]. Additionally, further studies are needed to elucidate the EMT-related mechanisms involved in the combined treatment with FPZ and DDP. In particular, changes in the expression of additional EMT markers, such as Slug and N-cadherin, should be examined to better understand the molecular basis of the observed effects.

DDP has been reported to induce intrinsic apoptosis through a mitochondria-dependent pathway in various malignancies [46,47]. In the present study, co-treatment with FPZ and DDP resulted in upregulation of Bax protein expression and increased levels of both cytosolic and mitochondrial ROS in gastric cancer cell lines, suggesting enhanced activation of the intrinsic apoptotic signaling pathway. Collectively, these findings suggest that increased ROS generation may be associated with the enhanced anticancer effects induced by co-treatment with FPZ and DDP. However, as ROS scavengers or ROS inhibition experiments were not included in the present study, the precise contribution of ROS to the observed effects remains unclear. In addition, ROS have been implicated in various biochemical alterations in cancer cells, including cell cycle arrest, programmed cell death, and autophagy [48,49]. Therefore, further studies employing ROS inhibitors are warranted to clarify the relationship between FPZ treatment and ROS upregulation, as well as the potential involvement of ROS in the diverse cellular responses induced by co-treatment with FPZ and DDP.

Unlike conventional 2D culture conditions, the in vivo microenvironment possesses a 3D architecture that restricts drug penetration and is involved in the acquisition of chemoresistance [50]. The current study demonstrated that the combined treatment with FPZ and DDP exerted enhanced anticancer effects in a 3D spheroid model, suggesting that this combination may retain its anticancer activity under conditions that more closely mimic the in vivo tumor architecture. Drug resistance commonly arises through multifaceted mechanisms, including cytokine signaling and increased expression of adenosine triphosphate (ATP)-binding cassette transporters [51,52]. Specifically, inflammatory cytokine-mediated autocrine loops play a crucial role in the acquisition and maintenance of chemoresistance across various cancer types, including gastric cancer [53,54,55]. In the present study, FPZ differentially modulated IL-6 and IL-8 mRNA expression depending on the gastric cancer cell line and attenuated DDP-induced inflammatory cytokine expression, particularly in MKN-45 cells. These findings suggest that the enhanced anticancer effects of the FPZ and DDP combination may be associated with the suppression of inflammatory cytokine induction; however, a direct role of these cytokines in chemoresistance was not established in the present study.

In drug repositioning, the evaluation of unexpected in vivo toxicity and confirmation of therapeutic efficacy under physiological conditions are indispensable prerequisites [56,57]. Accordingly, we investigated the in vivo effects of FPZ using a xenograft mouse model established with MKN-45 cells. No significant body weight loss was observed in the group treated with FPZ alone, whereas a marked reduction in body weight was observed in the DDP-treated groups. In line with previous studies reporting DDP-associated body weight loss [58,59], the observed reduction in body weight is likely to be primarily associated with DDP toxicity rather than FPZ treatment. Although FPZ monotherapy did not produce a significant antitumor effect in vivo, the combined treatment with FPZ and DDP significantly reduced both tumor volume and weight. The current findings suggest that FPZ potentiates the antitumor efficacy of DDP, potentially through mechanisms associated with chemoresistance. In this context, further investigations into alterations in drug resistance-related proteins and gene expression in tumor tissues will be necessary to clarify the underlying molecular mechanisms. In addition, no significant differences in liver weight were observed among the treatment groups. Nevertheless, comprehensive histopathological and functional analyses remain necessary to exclude tissue-specific toxicity or organ dysfunction. Moreover, given that FPZ acts as a DRD2 antagonist, further studies evaluating potential neurotoxicity are warranted [60,61]. As a first-generation antipsychotic, FPZ is associated with clinically relevant adverse effects, particularly extrapyramidal symptoms, including akathisia, dystonia, and parkinsonism, as well as tardive dyskinesia [62,63]. These known adverse effects should therefore be carefully considered when repurposing FPZ as an adjuvant anticancer agent. Additionally, the potential therapeutic benefits of enhancing DDP efficacy with FPZ should be carefully weighed against the risks associated with FPZ and any additional toxicity that may arise from the combination treatment. Accordingly, a comprehensive risk–benefit assessment is warranted to determine the feasibility of this drug repurposing strategy.

Beyond these considerations, several additional limitations of the present study should be acknowledged, including the use of a limited number of gastric cancer cell lines, a single xenograft model, and the lack of direct evaluation of potential ROS-associated toxicity of FPZ and DDP co-treatment in normal cells. Therefore, future studies should validate these findings in additional gastric cancer models and further clarify the molecular mechanisms underlying the enhanced anticancer effects of FPZ and DDP. In particular, the potential ROS-associated toxicity of FPZ and DDP co-treatment in normal cells should be investigated.

Despite these limitations, the present study demonstrated that FPZ enhanced the anticancer effects of DDP in gastric cancer models. Co-treatment with FPZ and DDP was associated with increased apoptotic signaling, elevated ROS levels, and suppression of EMT-related and pro-inflammatory responses in gastric cancer cells. In addition, the enhanced anticancer effects of FPZ and DDP were observed in both 3D spheroid and xenograft mouse models, supporting the potential of FPZ as an adjuvant to DDP-based chemotherapy in gastric cancer. Collectively, these findings highlight the potential of repurposing FPZ as a strategy to enhance the anticancer efficacy of conventional chemotherapy.

## 4. Materials and Methods

### 4.1. Chemicals

FPZ hydrochloride (Cat. No. PHR1792) and DDP (Cat. No. 232120) were purchased from Sigma-Aldrich (St. Louis, MO, USA). The FPZ and DDP were dissolved in distilled water and freshly prepared immediately prior to each experiment.

### 4.2. Cell Culture

Gastric cancer cell lines (MKN-45, Cat. No. 80103; AGS, Cat. No. 21739) were purchased from the Korean Cell Line Bank (KCLB; Seoul, Republic of Korea), and the human cardiomyocyte cell line AC16 (Cat. No. CRL-3568) was purchased from the American Type Culture Collection (ATCC; Manassas, VA, USA). MKN-45 cells were cultured in Roswell Park Memorial Institute 1640 medium (RPMI; Cat. No. M30450, R&D Systems, Minneapolis, MN, USA). AGS and AC16 cells were cultured in Dulbecco’s Modified Eagle’s Medium (DMEM; Cat. No. M22250, R&D Systems). All culture media were supplemented with 10% fetal bovine serum (FBS; Cat. No. F4135, Sigma-Aldrich), 10 mM 4-(2-hydroxyethyl)-1-piperazineethanesulfonic acid (HEPES; Cat. No. H0887, Sigma-Aldrich), and 1% Antibiotic-Antimycotic solution (Cat. No. A5955, Sigma-Aldrich). Subculture was performed using 0.05% Trypsin-EDTA (Cat. No. 25300054, Life Technologies, Carlsbad, CA, USA) at a split ratio of 1:5. Cells were maintained at 37 °C in a humidified incubator with 5% CO_2_.

### 4.3. Cell Viability Assay

MKN-45 (8 × 10^3^ cells/well), AGS (6 × 10^3^ cells/well), and AC16 (6 × 10^3^ cells/well) cells were seeded into 96-well plates and incubated for 24 h. The medium was then replaced with fresh medium containing 5% FBS (Control) or 5% FBS medium supplemented with either FPZ or DDP at various concentrations to determine their half-maximal inhibitory concentration (IC_50_) values. After 24 h of treatment, the medium was removed and replaced with 10% water-soluble tetrazolium salt (WST-1; Cat. No. CELLPRO-RO, Sigma-Aldrich) solution, followed by incubation for 30 min, as previously described [64]. To evaluate the combination effect of FPZ with DDP, MKN-45 (8 × 10^3^ cells/well) and AGS (6 × 10^3^ cells/well) cells were seeded into 96-well plates and incubated for 24 h. The medium was replaced with fresh 5% FBS medium containing vehicle (Control), FPZ (6 μM), DDP (at a concentration equivalent to 0.5 × IC_50_), or their combination. After 24 h of treatment, the medium was removed and replaced with 10% WST solution, followed by incubation for 30 min. The absorbance was measured at 450 nm using a Synergy Neo2 Hybrid Multimode Reader (Agilent, Santa Clara, CA, USA).

### 4.4. Trans-Well Migration Assay

The transwell migration assay was performed as previously described [65], with minor modifications. MKN-45 (5 × 10^5^ cells/well) and AGS (1 × 10^5^ cells/well) cells were seeded into the upper chamber of a transwell insert (8.0 μm membrane pore size; Cat. No. 3422, Corning, Inc., Corning, NY, USA). The upper chamber was filled with fresh 1% FBS media containing vehicle (Control), FPZ (6 μM), DDP (at a concentration equivalent to 0.5 × IC_50_), or their combination. The lower chamber was filled with 10% FBS medium to induce chemotactic migration. After 24 h of incubation, cells on the upper surface of the membrane were removed using a cotton swab, and migrated cells on the lower surface in the upper chamber were fixed with 4% PFA and stained with 0.5% crystal violet for 30 min. The number of migrated cells was quantified using ImageJ software (version 1.53; National Institutes of Health, Bethesda, MD, USA).

### 4.5. Annexin V and Propidium Iodide (PI) Double Staining

MKN-45 (5 × 10^5^ cells/well) and AGS (1 × 10^5^ cells/well) cells were seeded into 6-well plates. Once the cells reached approximately 70% density, the medium was replaced with fresh 5% FBS medium containing vehicle (Control), FPZ (6 μM), DDP (at a concentration equivalent to 0.5 × IC_50_), or their combination. After 24 h of treatment, apoptotic cells were stained using the Dead Cell Apoptosis Kit with Annexin V and PI for flow cytometry (Cat. No. V13241, Thermo Fisher Scientific, Waltham, MA, USA) according to the manufacturer’s instruction, with reference to a previously described Annexin V/PI-based apoptosis detection method [66]. Stained cells were detected using a BD Symphony flow cytometer (BD Biosciences, San Jose, CA, USA), and the apoptotic cell population was quantified using FlowJo software (version 10.8.1; FlowJo, LLC, Ashland, OR, USA).

### 4.6. 2′,7′-Dichlorodihydrofluorescein Diacetate (DCF-DA) Staining

MKN-45 (8 × 10^3^ cells/well) and AGS (6 × 10^3^ cells/well) cells were seeded into 96-well black-walled clear-bottom plates. After 24 h, the medium was replaced with fresh 5% FBS medium containing vehicle (Control), FPZ (6 μM), DDP (at a concentration equivalent to 0.5 × IC_50_), or their combination. After treatment, the medium was removed, and the cells were stained with 5 μM DCF-DA (Cat. No. D6883, Sigma-Aldrich) and 2 μM Hoechst 33342 (Cat. No. 62249, Thermo Fisher Scientific) for 30 min. Following three washes with phosphate-buffered saline (PBS), fluorescence was detected using the Lionheart™ FX Automated Microscope (BioTek Instruments, Winooski, VT, USA), with DAPI and GFP channels used to detect Hoechst 33342 and DCF-DA, respectively. Cytosolic reactive oxygen species (ROS) levels were quantified as the ratio of DCF-DA fluorescence intensity to the number of cells, following a previously described method [67].

### 4.7. MitoSOX^TM^ Staining

MKN-45 and AGS cells were seeded into 96-well black plates and treated under the same conditions as those used for the DCF-DA staining assay. After 24 h of treatment, cells were stained with 5 μM MitoSOX™ Red (Cat. No. M36007, Thermo Fisher Scientific) and 5 μM Hoechst 33342 for 10 min using a previously described method with minor modifications [68]. Following three washes with PBS, fluorescence was detected using a Lionheart™ FX Automated Microscope (BioTek Instruments). Mitochondrial ROS levels were quantified as the ratio of MitoSOX™ fluorescence intensity to the number of cells. Hoechst 33342 and MitoSOX™ fluorescence were detected using DAPI and RFP channels, respectively.

### 4.8. Spheroid Culture Assay

The spheroid culture assay was performed as previously described [69], with modifications. MKN-45 and AGS cells were seeded at a density of 4 × 10^3^ cells/well into 96-well clear round-bottom ultra-low attachment microplates (Cat. No. 7007, Corning, Inc., Corning, NY, USA). After one week of culture, the medium was replaced with fresh 5% FBS medium containing vehicle (Control), FPZ (6 μM), DDP (at a concentration equivalent to 0.5 × IC_50_), or their combination. After 24 h, spheroids were stained with Hoechst 33342 (20 μg/mL) and PI (50 μg/mL, Cat. No. P1304MP, Thermo Fisher Scientific) for 30 min. Fluorescence signals were detected using a Lionheart™ FX automated microscope (BioTek Instruments).

### 4.9. Reverse Transcription-Quantitative Polymerase Chain Reaction (RT-qPCR)

MKN-45 and AGS cells were cultured until they reached approximately 70% confluence. The medium was replaced with fresh 5% FBS medium containing vehicle (Control), FPZ (6 μM), DDP (at a concentration equivalent to 0.5 × IC_50_), or their combination. After 24 h, total RNA was extracted using TRIzol Reagent (Invitrogen, Carlsbad, CA, USA), and cDNA was synthesized using the PrimeScript™ RT Reagent Kit (Cat. No. RR037A, Takara Bio, Inc., Shiga, Japan) according to the manufacturer’s protocol. The reverse transcription reaction was performed in a final volume of 10 μL containing 2.0 μL of 5× PrimeScript Buffer, 0.5 μL of PrimeScript RT Enzyme Mix I, 0.5 μL of Oligo dT Primer, 0.5 μL of Random 6-mers, total RNA, and RNase-free water. Reverse transcription was performed at 37 °C for 15 min, followed by enzyme inactivation at 85 °C for 5 s. RT-qPCR was carried out using TB Green^®^ Premix Ex Taq™ II (Cat. No. RR820A, Takara Bio, Inc.) on the QuantStudio™ 1 Real-Time PCR System (Thermo Fisher Scientific, Waltham, MA, USA). Each reaction was performed in a final volume of 20 μL. The amplification conditions consisted of an initial denaturation at 95 °C for 30 s, followed by 40 cycles of 95 °C for 5 s and 60 °C for 30 s. A melting-curve analysis was performed after amplification to confirm amplification specificity. Relative mRNA expression levels were normalized to HPRT1 and calculated using the 2^−ΔΔCt^ method [70]. ΔCt was calculated as Ct(target gene) − Ct(*HPRT1*), and ΔΔCt was calculated as ΔCt(treatment group) − ΔCt(control group). Relative gene expression was expressed as 2^−ΔΔCt^. The primer sequences used for RT-qPCR are provided in Table S1.

### 4.10. Western Blot Assay

Western blot analysis was performed as previously described [71], with minor modifications. MKN-45 and AGS cells were cultured until they reached approximately 70% confluence. The medium was replaced with fresh medium containing 5% FBS and vehicle (Control), FPZ (6 μM), DDP (at a concentration equivalent to 0.5 × IC_50_), or their combination for 24 h. Total cellular proteins were extracted using PRO-PREP™ protein extraction solution (iNtRON Biotechnology, Seongnam, Republic of Korea) supplemented with a protease inhibitor cocktail (ATTO Corporation, Tokyo, Japan). Protein concentrations were determined using a bicinchoninic acid (BCA) protein assay following the Sigma-Aldrich BCA protein assay protocol, using BCA solution (Cat. No. B9643, Sigma-Aldrich) and 4% copper(II) sulfate solution (Cat. No. C2284, Sigma-Aldrich), with bovine serum albumin (BSA; Cat. No. A9418, MilliporeSigma, Burlington, MA, USA) used as the protein standard. Western blot analysis was performed by loading 30 μg of total protein in a final sample volume of 20 μL per lane. Total proteins were separated by SDS-PAGE on 8% gels for E-cadherin and 10% gels for Bax, followed by transfer onto polyvinylidene difluoride (PVDF) membranes (Amersham™ Hybond™ P; Cat. No. 10600023, Cytiva, Marlborough, MA, USA). The membranes were blocked with 5% BSA in Tris-buffered saline containing 0.1% Tween-20 (TBS-T) for 1 h 30 min at room temperature. After blocking, the membranes were incubated overnight at 4 °C with primary antibodies against Bax (1:1000; Cat. No. 2772, Cell Signaling Technology, Danvers, MA, USA), E-cadherin (1:1000; Cat. No. 3195, Cell Signaling Technology), and GAPDH (1:5000; Cat. No. ABS16, MilliporeSigma) diluted in 5% BSA in TBS-T (Cat. No. 11332465001, Roche Diagnostics, Basel, Switzerland). The membranes were then incubated with HRP-conjugated goat anti-rabbit IgG (1:3000; Cat. No. 1706515, Bio-Rad Laboratories, Hercules, CA, USA) or HRP-conjugated goat anti-mouse IgG (1:10,000; Cat. No. 1721011, Bio-Rad Laboratories) secondary antibodies for 1 h at room temperature. Protein bands were visualized using Amersham™ ECL™ Select Western Blotting Detection Reagent (Cat. No. RPN2235, Cytiva) and images were captured using a LuminoGraph II imaging system (ATTO Corporation). Band intensities were semi-quantified using ImageJ software (version 1.53) and normalized to GAPDH.

### 4.11. Gastric Cancer Xenograft

Five-week-old female athymic nude mice (Koat:Athymic NCr-nu/nu) were purchased from Koatech (Pyeongtaek, Republic of Korea) for in vivo experiments. All animal experiments were approved by the Institutional Animal Care and Use Committee (IACUC) of Chungbuk National University (Approval No. CBNUA-25-0152-03) and conducted in accordance with institutional guidelines for the care and use of laboratory animals. All in vivo experiments were designed, conducted, and reported in accordance with the ARRIVE 2.0 guidelines. The mice were acclimatized to the animal facility for one week before initiation of the experiment and housed in polycarbonate cages (five mice per cage) under controlled environmental conditions (22 ± 2 °C, 50 ± 10% relative humidity, and a 12-h light/12-h dark cycle). Wood-chip bedding was used, and standard laboratory chow and water were provided ad libitum. Tumor implantation and treatment-group design were performed with reference to a previous MKN-45 xenograft study [72], with modifications. MKN-45 cells (5 × 10^6^ cells in 100 μL of PBS) were subcutaneously injected into the right flank of each mouse. Tumor volume was calculated using the formula: Tumor volume = (length × width^2^)/2. When tumor volumes reached approximately 100 mm^3^, treatment was initiated, and the mice were allocated to four groups (*n* = 5 per group; total *n* = 20) using a body weight-based Z-arrangement randomization method: (1) vehicle control, (2) FPZ (4 mg/kg), (3) DDP (4 mg/kg), and (4) FPZ (4 mg/kg) + DDP (4 mg/kg). All treatments were administered via intraperitoneal injections in a volume of 100 μL. Treatment was administered every 3 days, with the order of treatment administration varied among the experimental groups at each dosing session. The group in which tumor volume measurements were initiated was also varied at each measurement time point to minimize potential bias. Cage locations were kept constant throughout the experimental period. Animals were regularly monitored throughout the experimental period for body weight, tumor burden, general appearance, mobility, and signs of pain or distress. Humane endpoint criteria were predefined as a tumor volume of approximately 1500 mm^3^, body weight loss of >15%, or severe clinical signs. Tumor weight measurements and statistical analyses were performed by investigators blinded to the treatment-group allocation.

### 4.12. Statistical Analysis

In vitro experiments were performed at least three times to ensure the reproducibility and consistency of the results. All data were analyzed with GraphPad Prism software (version 8.0.1; GraphPad Software, San Diego, CA, USA). In vitro data are expressed as mean ± standard deviation (SD), and in vivo data were quantified and expressed as mean ± standard error of the mean (SEM). For the spheroid culture assay and in vivo data, normality was assessed using the Shapiro–Wilk test prior to parametric statistical analysis. Depending on the experimental design, statistical significance was determined using an unpaired *t*-test, one-way analysis of variance (ANOVA), or two-way ANOVA, followed by Dunnett’s multiple comparisons test where appropriate. All statistical comparisons performed are indicated in the corresponding figure legends. A *p*-value < 0.05 was considered statistically significant.

## Data Availability

All data generated or analyzed in this study are included in the published article. Further inquiries can be directed to the corresponding authors.

## Supplementary Materials

The following supporting information can be downloaded at: https://www.mdpi.com/article/10.3390/ph19091373/s1, Table S1. Oligonucleotide sequences for PCR reaction.

## Abbreviations

Anti-Anti	Antibiotic–Antimycotic
Bax	Bcl-2-associated X protein
CSCs	Cancer stem cells
DA	Dopamine
DCF-DA	2′,7′-Dichlorodihydrofluorescein diacetate
DDP	Cisplatin
DMEM	Dulbecco’s Modified Eagle Medium
DRD2	Dopamine Receptor D2
EGF	Epidermal Growth Factor
EMT	Epithelial–Mesenchymal Transition
E-cadherin	Epithelial cadherin
FACS	Fluorescence-Activated Cell Sorting
FBS	Fetal Bovine Serum
FPZ	Fluphenazine
HEPES	4-(2-hydroxyethyl)-1-piperazineethanesulfonic acid
IACUC	Institutional Animal Care and Use Committee
IGF	Insulin-like Growth Factor
PI	Propidium Iodide
ROS	Reactive Oxygen Species
WST	Water-Soluble Tetrazolium
2D	Two-Dimensional
3D	Three-Dimensional
