# Supplementary material for: Anticancer Effects of Fluphenazine Alone and in Combination with Cisplatin in Gastric Cancer Cells and a Mouse Xenograft Model
Source: Pharmaceuticals (Basel). 2026 Aug 31;19(9):1373. doi: 10.3390/ph19091373 (PMC13610874; doi:10.3390/ph19091373)
Supplement: Supplementary file 1 [file pharmaceuticals-19-01373-s001.zip › pharmaceuticals-4474071-supplementary.pdf]

Table S1. Oligonucleotide sequences for PCR reaction

Gene	Primer	Sequence (5′→3′)
HPRT1	Forward	AATTCTTTGCTGACCTGCTG
	Reverse	TACTGCCTGACCAAGGAAAG
IL6	Forward	GCCCAGCTATGAACTCCTTCT
	Reverse	GAAGGCAGCAGGCAACAC
IL8	Forward	GGGTGGAAAGGTTTGGAGTATGT
	Reverse	CTTGGCAGCCTTCCTGATTTC
